# Supplementary material for: Social Behavioral Deficits in Krushinsky-Molodkina Rats, an Animal Model of Audiogenic Epilepsy
Source: J Pers Med. 2022 Dec 14;12(12):2062. doi: 10.3390/jpm12122062 (PMC9781841; doi:10.3390/jpm12122062)
Supplement: Supplementary file 1 [file jpm-12-02062-s001.zip › jpm-2095137-supplementary.pdf]

Table S1 Elevated plus maze

Parameters	KM stain (mean±Std. er. of mean)	Wistar stain (mean±Std. er. of mean)	p-value
Percentage of time spent in open arms	6.2±3.4	16.3±2.4	0.004
Percentage of time spent in closed arms	66.8±4.6	76.5±3.3	0.11
% of time spent in center	27.0±4.6	7.2±3.0	0.0002
Closed/open-arms entry ratio	3.3±0.8	3.3±0.8	0.76
Total number of entries in any arm	6.6±0.7	9.3±0.7	0.003
Visits to closed arms	5.8±0.7	6.5±0.6	0.28
Visits to open arms	0.8±0.3	2.8±0.4	0.001
Falls	0.1±0.1	0.1±0.1	0.94
The path length, mm	7227±617	19571±1726	0.00002
Number of rearings	12.3±1.2	20.4±1.9	0.006
Number of facial grooming	10.0±1.2	2.8±0.8	0.00003
Number of hole body grooming	1.9±0.3	1.7±0.4	0.37
Number of freezing episodes	20.5±2.0	10.6±2.1	0.001
Average freezing time, sec	13.5±3.5	7.9±1.5	0.007
Total freezing time, sec	212.2±28.9	84.5±26.6	0.0003
Stereotypy (head waiving)	1.5±0.3	0.6±0.3	0.052

Statistical significance is calculated using the criterion Mann-Whitney U-test. Significantly differing parameters are highlighted in color. Data are represented as the mean ± SEM

Table S2 Three-chambered social preference test

Parameters	KM stain (mean±Std. er. of mean)	Wistar stain (mean±Std. er. of mean)	p-value
The path length, mm	15836±2991	22226±2440	0.09
Path length in stimulus rat compartment, mm	4195±1343	10462±1175	0.0004
Path length in the empty cage compartment, mm	5411±1029	6049±781	0.56
Path length in the intermediate compartment, mm	6032±1339	5714±726	0.98
Time spent in stimulus rat compartment, sec	106.5±21.1	187.8±19.5	0.02
Time spent in the empty cage compartment, sec	317.6±47.0	173.2±16.8	0.009
Time spent in the intermediate compartment, sec	175.6±33.3	239.1±25.4	0.18
Number of rearings	9.1±1.9	14.0±1.8	0.09
Number of facial grooming	3.8±0.9	5.0±0.9	0.39
Number of hole body grooming	1.3±0.6	1.0±0.2	0.59
Number of contacts with stimulus rat	1.5±0.6	6.3±0.8	0.00003
Total contact time, sec	10.8±4.7	49.6±11.6	0.0005
Number of freezing episodes	17.7±3.8	9.1±1.8	0.06
Average freezing time, sec	9.5±1.4	5.6±0.3	0.052
Total freezing time, sec	188.7±46.7	56.9±13.7	0.04
Stereotypy (head waiving)	1.4±0.7	0.8±0.4	0.80
Sociability index	0.51±0.12	1.38±0.27	0.01

Statistical significance is calculated using the criterion Mann-Whitney U-test. Significantly differing parameters are highlighted in color. Data are represented as the mean ± SEM

Table S3 Three-chambers social novelty test

Parameters	*КМ stain (mean±Std. er. of mean)*	*Wistar stain (mean±Std. er. of mean)*	*p-value*
The path length, mm	4010±822	12568±1680	0.00004
Path length in "old" stimulus rat compartment, mm	608±305	3632±616	0.0003
Path length in the "new" stimulus rat compartment, mm	2034±475	5326±768	0.002
Path length in the intermediate compartment, mm	1574±457	3610±570	0.02
Time spent in stimulus rat compartment, sec	61.0±24.4	196.3±31.7	0.002
Time spent in the new stimulus rat compartment, sec	271.1±51.4	272.0±26.6	0.69
Time spent in the intermediate compartment, sec	271.4±50.9	131.7±15. 9	0.0003
Number of rearings	1.4±0.5	8.6±1.3	0.00002
Number of facial grooming	1.8±0.7	4.1±1.0	0.06
Number of hole body grooming	0.0±0.0	1.4±0.2	0.0001
Number of contacts with "old" stimulus rat	0.3±0.1	2.7±0.3	0.000001
Number of contacts with "new" stimulus rat	0.8±0.3	4.1±0.7	0.001
Total "old" rat contact time, sec	61.017±0.8	196.30±7.6	0.00
Total "new" rat contact time, sec	8.6±3.2	48.8±8.7	0.001
Number of freezing episodes	19.3±3.2	18.2±2.4	0.66
Average freezing time, sec	59.4±23.9	6.9±0.6	0.00
Total freezing time, sec	467.6±20.1	135.2±27.1	0.00
Stereotypy (head waiving)	0.9±0.4	0.6±0.2	0.83

Statistical significance is calculated using the criterion Mann-Whitney U-test. Significantly differing parameters are highlighted in color. Data are represented as the mean ± SEM

Table S4 Comparison between three-chambered social preference test and three-chambered social novelty test for KM stain

Parameters	*3-chambered social preference (mean±Std. er. of mean)*	*3-chambered social novelty (mean±Std. er. of mean)*	*p-value*
Time spent in stimulus rat compartment, sec	106.5±21.1	61.0±24.4	0.061
Time spent in the empty/new stimulus rat compartment, sec	317.6±47.0	271.1±51.4	0.48
Time spent in the intermediate compartment, sec	175.6±33.3	271.4±50.9	0.15
Number of rearings	9.1±1.9	1.4±0.5	0.005
Number of facial grooming	3.8±0.9	1.8±0.7	0.11
Number of long grooming	1.3±0.6	0.0±0.0	0.043
Number of contacts with "old" stimulus rat	1.5±0.6	0.3±0.1	0.11
Number of contacts with "new" stimulus rat Comparison to Number of contacts with "old" stimulus rat	1.5±0.6	0.8±0.3	0.36
Total "old" rat contact time, sec	10.8±4.7	61.017±0.8	0.042
Total contact time with "new" stimulus rat Comparison to Number of contacts with "old" stimulus rat, sec	10.8±4.7	8.6±3.2	0.72
Number of freezing episodes	17.7±3.8	19.3±3.2	0.72
Average freezing time, sec	9.5±1.4	59.4±23.9	0.002
Total freezing time, sec	188.7±46.7	467.6±20.1	0.004
Stereotypy (head waiving)	1.4±0.7	0.9±0.4	0.5

Statistical significance is calculated using the criterion Wilcoxon Matched pairs test. Significantly differing parameters are highlighted in color. Data are represented as the mean ± SEM

Table S5 Comparison between three-chambered social preference test and three-chambered social novelty test for Wistar stain

Parameters	*3-chambered social preference (mean±Std. er. of mean)*	*3-chambered social novelty (mean±Std. er. of mean)*	*p-value*
Time spent in stimulus rat compartment, sec	187.8±19.5	196.3±31.7	0.87
Time spent in the empty|new stimulus rat compartment, sec	173.2±16.8	272.0±26.6	0.007
Time spent in the intermediate compartment, sec	239.1±25.4	131.7±15. 9	0.01
Number of rearings	14.0±1.8	1.4±0.5	0.03
Number of facial grooming	5.0±0.9	1.8±0.7	0.44
Number of long grooming	1.0±0.2	0.0±0.0	0.003
Number of contacts with "old" stimulus rat	6.3±0.8	2.7±0.3	0.002
Number of contacts with "new" stimulus rat Comparison to Number of contacts with "old" stimulus rat	6.3±0.8	4.1±0.7	0.04
Total "old" rat contact time, sec	49.6±11.6	196.30±7.6	0.5
Total contact time with "new" stimulus rat Comparison to Number of contacts with "old" stimulus rat, sec	49.6±11.6	48.8±8.7	0.8
Number of freezing episodes	9.1±1.8	18.2±2.4	0.005
Average freezing time, sec	5.6±0.3	6.9±0.6	0.17
Total freezing time, sec	56.9±13.7	135.2±27.1	0.008
Stereotypy (head waiving)	0.8±0.4	0.6±0.2	0.6

Statistical significance is calculated using the criterion Wilcoxon Matched pairs test. Significantly differing parameters are highlighted in color. Data are represented as the mean ± SEM
